# Editorial: Mechanisms of dysregulated antibody responses in inborn errors of immunity

**DOI:** 10.3389/fimmu.2023.1335217

**Published:** 2023-11-21

**Authors:** Qing Min, Jolan E. Walter, Harry W. Schroeder, Peter D. Burrows, Ji-Yang Wang

**Affiliations:** ^1^ Department of Clinical Immunology, Children’s Hospital of Fudan University, National Children’s Medical Center, Shanghai, China; ^2^ Division of Pediatric Allergy/Immunology, University of South Florida at Johns Hopkins All Children’s Hospital, St. Petersburg, FL, United States; ^3^ Division of Clinical Immunology and Rheumatology, Department of Medicine, University of Alabama at Birmingham Heersink School of Medicine, Birmingham, AL, United States; ^4^ Department of Microbiology, University of Alabama at Birmingham, Birmingham, AL, United States; ^5^ Department of Immunology, School of Basic Medical Sciences, Fudan University, Shanghai, China; ^6^ Department of Microbiology and Immunology, College of Basic Medical Sciences, Zhengzhou University, Zhengzhou, China; ^7^ Shanghai Huashen Institute of Microbes and Infections, Shanghai, China

**Keywords:** dysregulated antibody response, partial RAG deficiency, IRF4, immune dysregulation, XLA

Human Inborn Errors of Immunity (IEI) represent a diverse array of genetic disorders affecting the immune system and result in increased susceptibility to infections, autoinflammation, autoimmunity, allergies, and/or malignancies (1, 2). To date, a total of 485 genetic defects that cause IEI have been identified (3), with over two-thirds of them being associated with dysregulated antibody production (4). Primary antibody deficiencies (PAD) are characterized by significantly reduced serum levels of one or more immunoglobulin (Ig) classes (4, 5). There are also patients who display normal serum Ig levels yet fail to produce specific antibodies against pathogens (5). Moreover, some patients with IEI produce excessive amounts of Igs, including autoantibodies (6, 7). Such dysregulated antibody production can be caused by molecular defects intrinsic to B-cells or abnormalities in other cell types, resulting in impaired B cell development, maturation, survival, activation, Ig gene class switching, or plasma cell differentiation. Understanding the cellular and molecular basis of the dysregulated antibody production should benefit the diagnosis and treatment of IEI associated with abnormal antibody levels. This Research Topic features three review articles focusing on dysregulated antibody responses in both combined immunodeficiency and immune dysregulation, and one original study presenting a comprehensive analysis of the clinical, immunological, and genetic features of X-linked agammaglobulinemia (XLA) patients in Malaysia.

The recombination activating genes (RAG1 and RAG2) are essential not just for Ig gene rearrangements but also for receptor editing, a mechanism pivotal for eliminating autoreactive B cells. Complete absence of RAG1 or RAG2 leads to severe combined immunodeficiency (SCID) with no T and B cells. In contrast, partial RAG deficiency (pRD) results in combined immunodeficiency (CID) associated with immune dysregulation, including autoantibody production. Min et al. provide an in-depth analysis of the clinical and immunological characteristics in a cohort of 20 pRD patients. Their article highlights the reduced B cell repertoire diversity, compromised receptor editing, increased homeostatic proliferation of peripheral B cells, and enhanced plasma cell differentiation among these patients. They propose a model for the potential mechanism underlying autoantibody production in pRD patients (**Figure 1**). According to this model, insufficient RAG activity hampers receptor editing, allowing some autoreactive B cells to escape from the bone marrow into the periphery. These cells then undergo homeostatic proliferation driven by elevated levels of BAFF in the lymphopenic environment. This expansion triggers phenotypic and functional changes in the B cells, notably producing CD27^-^IgD^-^ double negative (DN) B cells, CD11c^high^Tbet^+^ cells, or CD27^+^IgD^-^ memory-like B cells. Upon activation by TLR ligands and cytokines or in response to self or foreign antigens, these altered B cells can efficiently differentiate into antibody-secreting cells, leading to the robust production of antibodies, including autoantibodies. Importantly, Min et al. found a strong correlation between the decreased number and proportion of naïve B cells and the increased proportions of DN B and memory-like B cells after analyzing 52 patients with IEI caused by other genetic mutations or with unknown causative genes (7). Specifically, they discovered that when the absolute B cell count drops below 100/µl in the peripheral blood, both DN B and the memory-like B cells increase drastically. Therefore, the generation of these abnormal B cell subsets is likely a general feature in patients with B cell lymphopenia.

**Figure 1:**
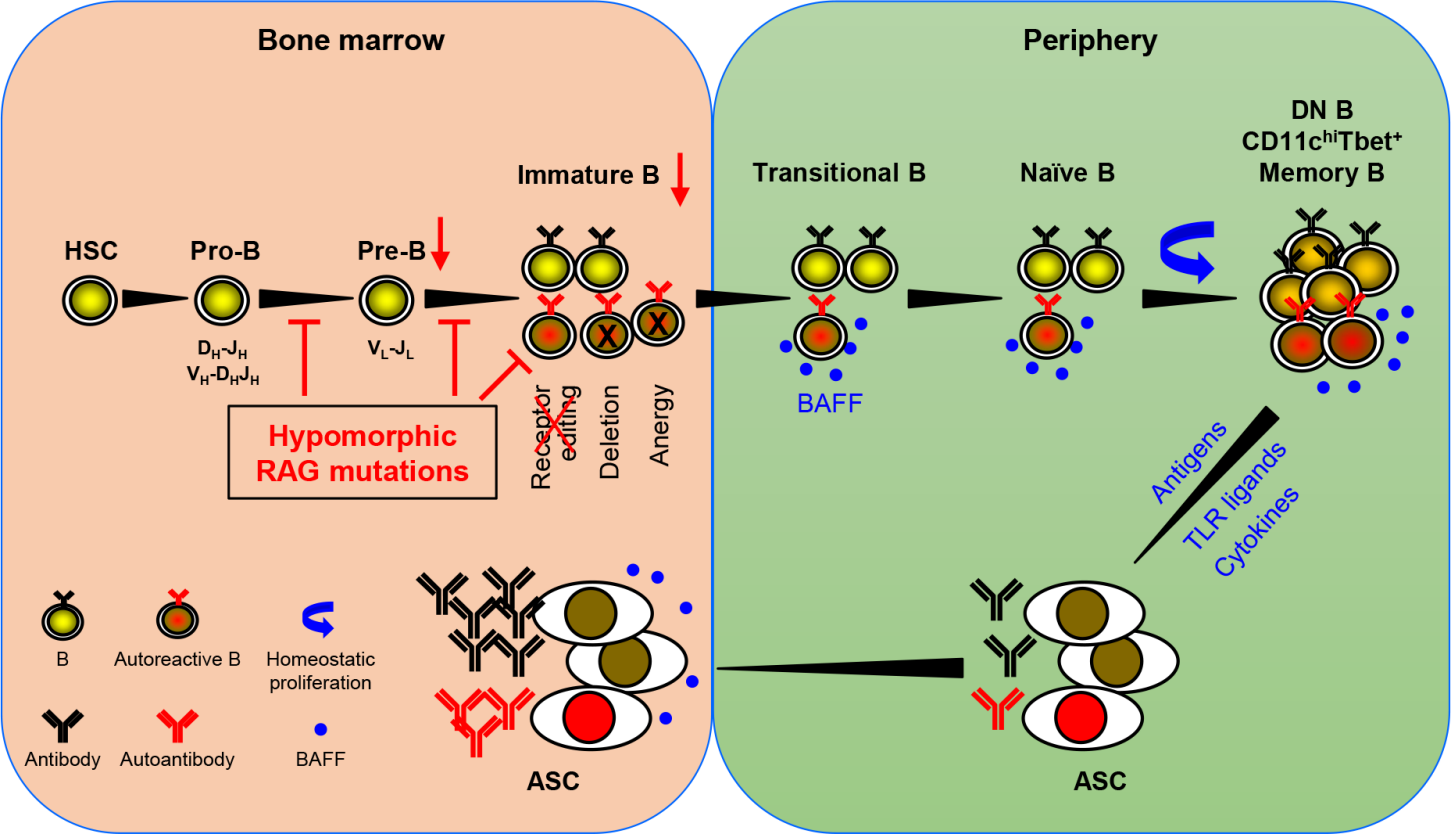
A schematic representation of autoantibody production in patients with pRD. pRD leads to limited V(D)J recombination, causing an incomplete block in B cell development at the pro-B to pre-B and pre-B to immature B stages. This results in a reduced population of pre-B and immature B cells (indicated by red arrows). Moreover, pRD disrupts receptor editing and allows some autoreactive immature B cells to leave BM and become transitional B (TrB) cells in the periphery. These autoreactive TrB can survive due to elevated levels of BAFF in the lymphopenic environment and further become mature naïve B cells. These naïve B cells undergo homeostatic proliferation and differentiate into CD27^-^IgD^-^ DN B, CD11c^hi^Tbet^+^ B or CD27^+^IgD^-^ memory-like B cells. It is noteworthy that these DN B, CD11c^hi^Tbet^+^ B and memory-like B cells may not represent distinct subsets but rather populations with overlapping features. These more differentiated B cells can efficiently become ASCs upon stimulation with TLR ligands, cytokines, or antigenic stimulation, and migrate to the BM. Elevated serum BAFF levels may further facilitate the formation and survival of ASCs.

The interferon regulatory factor (IRF) family members are important for immune cell development and functions. Thouenon and Kracker have reviewed the phenotypes and pathophysiological mechanisms associated with IEI resulting from mutations in IRF4 and other IRFs. While a multitude of somatic mutations in IRF4 have been identified in hematopoietic cancers, only four cases of immune disorders have been linked to IRF4 mutations. Consistent with earlier findings in mice (8), homozygous loss-of-function (LOF) mutations in IRF4 lead to a profound CID. In contrast, a heterozygous LOF R98W mutation is associated with Whipple’s disease, an inflammatory disorder in the intestine, with age-dependent incomplete penetrance and without causing severe immunodeficiency. An autosomal dominant F359L variant displaying neomorphic effects disrupts IRF4 transcriptional activity, leading to CID with hypogammaglobulinemia in three patients. An autosomal dominant T95R variant exhibits a simultaneous multimorphic combination of loss, gain, and new functions for IRF4 resulting in early-onset CID in seven patients with agammaglobulinemia (9). However, the precise mechanisms by which F359L and T95R heterozygous mutations cause CID remain to be elucidated. In addition, Thouenon and Kracker provide an overview of variants in other IRF family members that are implicated in IEI.

Patients with immunodeficiencies often experience immune dysregulation, leading to autoinflammation, autoimmune diseases and an increased risk of malignancies. Recognizing the unique challenges posed by these conditions, “Disease of immune dysregulation” has been established as an independent category of IEI by the International Union of Immunological Societies (IUIS). Ren et al. have contributed a comprehensive review detailing the genetic alterations, clinical symptoms and therapeutic approaches for a range of newly identified primary immune dysregulation diseases. This includes disorders arising from deficiencies in SLC7A7, CD122, DEF6, FERMT1, SOCS1, TGFβ1, RIPK1, CD137, and TET2. Among these, patients deficient in CD122, DEF6, SOCS1, CD137, and TET2 present with autoimmune hemolytic anemia (AIHA). Moreover, CD122 deficiency leads to elevated serum Igs, while patients lacking SOCS1 or CD137 have reduced serum Igs. However, the underlying mechanisms leading to such aberrant antibody production are not fully understood and warrant further investigation.

In the study by Chear et al., the demographic, clinical and immunological phenotypes and genetic characteristics of XLA within the Malaysian population are assessed. Twenty-two patients from 16 unrelated families are included in the study. Over 90% of these patients display very low levels of serum IgG and IgA. The most frequent clinical presentation is pneumonia (n=13), followed by otitis media (n=12). Two patients present with normal Ig levels; however, it is unclear whether these two patients also have an increased presence of CD27^-^IgD^-^ DN B cells, CD11c^high^Tbet^+^ cells, or memory-like B cells in their peripheral blood. The gene encoding Bruton’s tyrosine kinase (BTK) is mutated in XLA and this study also underscores the efficacy of using flow cytometric analysis of BTK protein expression in monocytes as a rapid and useful diagnostic method for identifying XLA patients and carriers.

These review papers and the original research article encompassed in this Research Topic offer important and timely insights into the mechanisms of dysregulated antibody responses in conditions such as partial RAG deficiency, IRF4 deficiency, immune dysregulation diseases, and XLA in Malaysia. The collective insights from these contributions are significant, as they help advance diagnostic accuracy and aid in the optimization of therapeutic strategies.

## Author contributions

QM: Data curation, Funding acquisition, Investigation, Project administration, Writing – original draft. JW: Conceptualization, Supervision, Validation, Writing – review & editing. HS: Conceptualization, Supervision, Writing – review & editing. PB: Conceptualization, Supervision, Writing – review & editing. J-YW: Conceptualization, Funding acquisition, Methodology, Project administration, Supervision, Writing – review & editing.
